# Could behaviour change interventions be incorporated into cardiac rehabilitation programmes for insomnia and poor sleep quality management? A scoping review

**DOI:** 10.1007/s11325-026-03707-x

**Published:** 2026-05-23

**Authors:** Elizabeth White, Hady Atef

**Affiliations:** School of Allied Health Professions and Pharmacy (SAHPaP), Keele University, Staffordshire, UK

**Keywords:** Sleep, Cardiac rehabilitation, Cardiovascular disease

## Abstract

**Background:**

Cardiovascular disease (CVD) is caused by modifiable and non-modifiable risk factors and is consistently recognised as a leading cause of morbidity and mortality. Poor sleep caused by sleep disorders such as insomnia are linked to increased mortality in patients with CVD, yet it is routinely overlooked as a cause to treat. Furthermore, despite the impact of sleep disorders, management strategies are notably underrepresented in cardiac care guidelines and cardiac rehabilitation.

**Aim of review:**

This scoping review aimed to assemble recent published literature to explore how behaviour change interventions could be utilised as a method to integrate sleep management strategies into state-of-the-art cardiac rehabilitation for patients with cardiovascular disease.

**Methods:**

Four medical databases (Cochrane Library, MEDLINE, PsycINFO and CINAHL+) were searched using a PICO framework, publishing a total of 782 results. Of the 782 records identified, 23 were included to inform this review, including an additional article found from bibliography searching. A variety of study designs were identified, including randomised controlled trials, pilot trials, secondary data analysis and systematic reviews.

**Results:**

Cognitive behavioural therapy emerged as the most consistently effective intervention, particularly in group and web-based formats. Brief behavioural treatment for insomnia was also identified as an emerging flexible and easily adaptable approach, also producing promising reductions in insomnia severity.

**Conclusions:**

Behaviour change interventions demonstrate significant potential for improving sleep outcome measures and insomnia severity when tested among patients living with CVD. Evidence gaps include heterogeneity in intervention protocols and sample populations. Future studies must involve key stakeholders to better shape the suggested interventions. Also, they should consider this whilst exploring the role of tele-health and the need for flexibility for strategies to be implemented into cardiac rehabilitation.

**Supplementary Information:**

The online version contains supplementary material available at 10.1007/s11325-026-03707-x.

## Highlights

Cognitive Behavioural Therapy For Insomnia (CBTI) and Brief Behavioural Treatment For Insomnia (BBTI) are scalable and effective tools for improving sleep in CR.

Web-based delivery enhances accessibility and adherence to sleep interventions in CR.

Future trials are needed to embed sleep into CR using behavioural change models.

**Graphical abstract:**

**Supplementary Information:**

The online version contains supplementary material available at 10.1007/s11325-026-03707-x.

## Graphical abstract

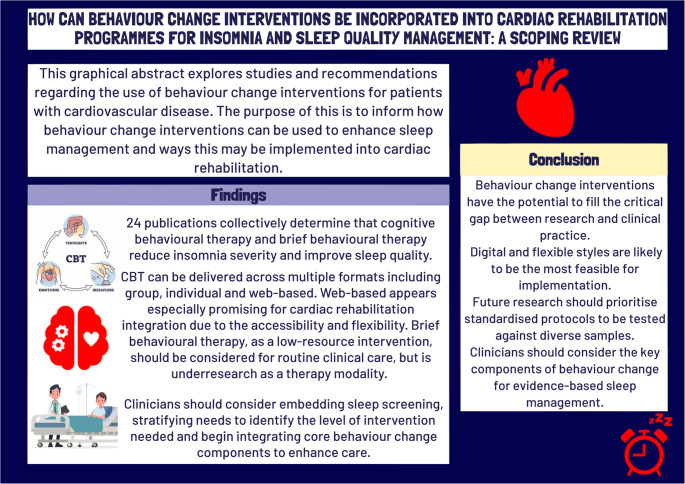

## Introduction

Cardiovascular disease (CVD) remains a leading cause of infirmity and mortality in the United Kingdom (UK), particularly among the ageing and sedentary population, where its prevalence continues to rise [1]. Alongside being a significant contributor to mortality, CVD is reported to cost the UK £28 billion in healthcare fees, incorporating long-term care and disability investments [2]. This collective title covers all types of multifactorial diseases that may affect the cardiovascular system, encompassing atherosclerosis, peripheral artery disease, heart failure (HF), myocardial infarction (MI), and rhythmic disorders [3]. It is universally understood that modifiable risk factors, such as hypertension, smoking, and physical inactivity, contribute to the development of CVD [4]. There are also non-modifiable risk factors, including older age, ethnicity, sex, and family predisposition, which may further increase the potential development of CVD [1].

The American Heart Association (AHA) established the ‘Life Essential 8’ in 2022 as the key measure for maintaining or enhancing cardiovascular health [5]. Within this, healthy sleep is recognised as a core health behaviour due to the associated effects of vascular healing, improving immunity and psychological health, and brain function enrichment [5]. A meta-analysis produced by Lui et al. [6] in 2020 uncovered that disrupted sleep and insomnia are significant contributors to cardiovascular morbidity due to their impact on multiple physiological systems. Unregulated sleep was also found to cause a loss in CVD-free years [7], supported by Zhang et al. [8], who identified that up to 30% of CVD patients experienced insomnia.

The National Institute for Clinical Excellence (NICE) recommends an individualised and comprehensive approach to risk factor management for patients with CVD, placing emphasis on the importance of lifestyle modification [9]. Cardiac rehabilitation (CR) is recognised as a strategy to improve cardiac function and reduce further risk through diet and health education, exercise, and stress management, with a focus on improving health behaviours and quality of life [10]. The programme usually consists of three phases: phase one being an inpatient stay, with phase two transitioning to a supervised mobility programme within an outpatient setting, and finally concluding with lifestyle modification and exercise training to ensure lifetime maintenance of reduced risk [11]. Despite the strong association between the impacts of unregulated sleep on cardiovascular health, sleep management strategies are routinely ignored within rehabilitation protocols, including the British Association of Cardiopulmonary Rehabilitation (BACPR) Standards and core components guidelines [12]. The NICE guidelines of CR amongst coronary syndromes also exclude sleep management recommendations, producing a gap in established guidelines [13]. This omission is extremely noticeable when the impact of sleep quality on CVD is clear, as an improvement in sleep quality can facilitate cognitive and emotional advancement, thus possibly improving motivation and the ability to engage with an effective rehabilitation programme [14].

Ghane et al. reported that current sleep interventions are viewed as costly and time-consuming for health services, deterring their implementation within health services [15]. To combat this, emerging evidence suggests the use of health behaviour change [16]. Behaviour change (BC) techniques usually include effective lifestyle change interventions, such as diet control, increasing physical activity, and smoking cessation, with attention paid to reward, identity, and commitment to encourage longevity [17]. This is essential within an effective CR programme, which incorporates patient education alongside health behaviour analysis [18]. A theoretical understanding of BC techniques may maximise the potential for health behaviour change, thus supporting the use of techniques based on evidence-based models [19]. By utilising techniques based on behaviour change theory, sleep management may transition from reliance on clinicians, pharmaceutical modalities or physical items such as eye masks [20] to self-management by supporting patients’ choices to engage with or avoid healthy sleep behaviours [21]. Emerging evidence regarding insomnia management considers how educational programmes and stress management may be useful in improving poor sleep behaviours and habits [22] by incorporating specific behaviour change frameworks, such as cognitive behavioural therapy (CBT). The use of cognitive behavioural therapy for CVD patients who suffer from insomnia has been regularly endorsed and recommended, but is yet to be recognised as an essential part of a holistic CR treatment [16].

## Purpose of the review

This scoping review will aim to expose published opinions and recommendations regarding the use of behaviour change interventions for improving insomnia and poor sleep quality for patients with CVD. Actionable recommendations may be made for readers and stakeholders to consider the most feasible and efficient intervention for the CVD population. In-depth insights will be provided by analysing the clinical improvements, engagement and flexibility of various behaviour change interventions to address the gap in the literature.

## Methodology

To inform the scope of this review and ensure relevance across a broad range of cardiac conditions and sleep disorders, a multidisciplinary research team engaged with key stakeholders in alignment with PRISMA-ScR [23]. These included CR participants (*n* = 9), all of whom reported insomnia and poor sleep quality as central concerns, and experienced CR clinicians (*n* = 6), comprising nurses (*n* = 2), a cardiologist (*n* = 1), exercise physiologists (*n* = 2), and a pharmacist (*n*= 1). Clinicians reported that sleep is not routinely assessed within standard CR pathways; typically, a single screening question related to positional sleep apnoea is used, without subsequent structured assessment for sleep-disordered breathing (SDB) or insomnia. All patient stakeholders (100%) reported insomnia in addition to poor sleep quality and indicated that they had not accessed sleep management strategies beyond basic sleep hygiene advice, which they perceived as ineffective. These stakeholder insights highlighted a clear gap in the assessment and management of insomnia and reduced sleep quality within CR services. Consequently, this scoping review undertook a comprehensive search and utilised an established methodological framework [24] to map and synthesise the existing evidence on the management of insomnia and poor sleep quality within CR settings.

The database search was conducted across MEDLINE, CINAHL+, PsychINFO and the Cochrane Library, all chosen with the purpose of gaining a variety of clinical trials and evidence reviews. Both MEDLINE and the Cochrane Library were chosen due to their broad coverage of biomedical literature, whereas CINAHL + was searched to identify articles particularly focused on allied health literature, and PsychINFO was searched due to the need for behavioural and psychological science reports. The full search strings conducted across each database can be seen in Appendix A.

The initial database search was conducted on 24 March 2025. This search identified 67 records from MEDLINE, 47 from APA PsycInfo, 18 from CINAHL Plus, and 650 from the Cochrane Library for title and abstract screening. To ensure currency of the evidence, the search was updated one year later on 29 March 2026. This updated search identified a further 24 records from MEDLINE, six from APA PsycInfo, four from CINAHL Plus, and 28 from the Cochrane Library. All records retrieved from the updated search were screened against the eligibility criteria and were excluded, as they did not meet the inclusion criteria for this review.

All articles identified across the four databases were transported into RefWorks, which provided a web-based bibliography which enhanced research productivity [24]. By utilising the tools provided, 88 duplicates across the databases were initially removed before screening. All titles and abstracts were reviewed, with 633 articles excluded as they were deemed irrelevant to the research title. Following this, investigations into the access to the articles sought for retrieval resulted in 32 exclusions due to a lack of published written results by government trials or restrictions on accessibility. This resulted in 29 articles suitable for assessment for eligibility against an established inclusion and exclusion criteria, with any further reasons for exclusion being reported. The inclusion criteria ensured all articles have been published after 2010 to assure the results are representative of a modern society, are published in English to avoid inconsistencies with evidence in translation and are not preliminary studies where no results have been published. Articles were included regardless of the type of CVD, severity, presentation and length of morbidity. Articles were also included regardless of age, gender, and ethnicity. The articles were excluded if there was no evidence of participants diagnosed with any form of CVD, if the study design did not utilise an established behaviour change intervention, or if the study did not include participants living with insomnia or poor sleep quality.

Following article selection, all bibliographies were examined with the intent to identify any further relevant articles. This process identified two additional articles, and it quickly became evident that the saturation points for new articles to appear had been met. Utilising stage four of the Arksey and O’Malley [24] framework, data charting involved extracting authors and year of publication, study location, study populations, intervention type and duration, outcome measures, and important results or conclusive thoughts. Categorisation of the data, informed by data extraction, provided a basis for in-depth analysis. Due to the aim of this review being to collate and provide insight into any interventions, rather than to assess the quality of evidence for a particular method, no quality appraisal of any articles was performed.

## Results

**Fig. 1:**
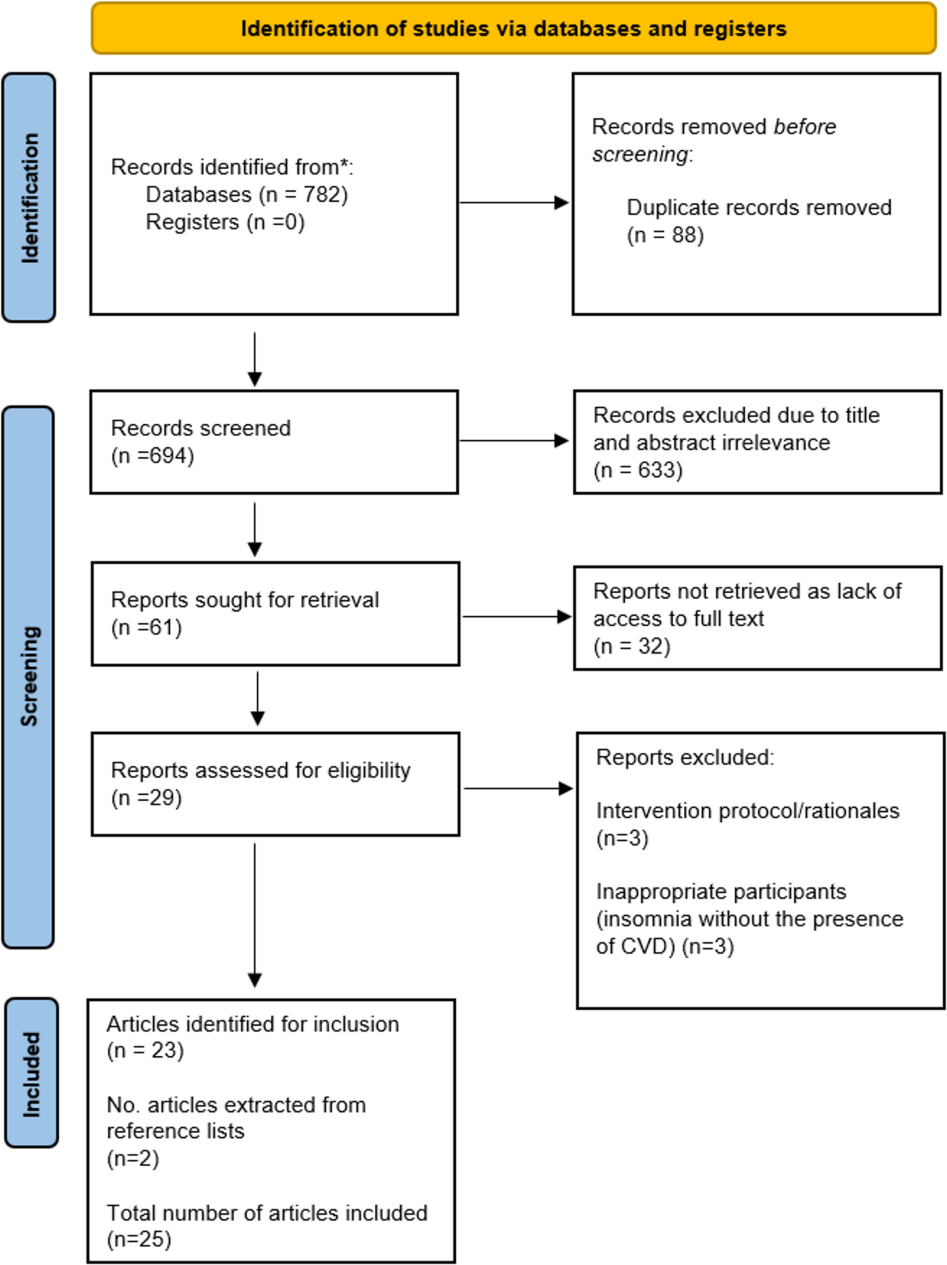
An image demonstrating the identification of studies process, outlined in a PRISMA chart

Across the four databases, 782 results were identified. Of the 747 results, 23 were deemed suitable for inclusion within the review, alongside one retrieved from reference list searching, resulting in 24 articles. Of the 23 collated from the database search, eight of those were from the Cochrane library, seven were from PsycINFO, five were from MEDLINE, and only three were from CINAHL+ (Fig. 1). Across the 24 results, 2,650 individual participants were accounted for. Study characteristics of the included results, particularly the details of the interventions delivered, can be seen in Table 1.

**Table 1:** A table demonstrating the study characteristics, sleep outcome measures, details of any intervention delivered, the location and interventionist (if not online), and the key findings of all included results

Study: author (year); country	Population	Sleep outcome measures	Intervention type, duration, session details		Key findings
Systematic review: Abdelaziz et al. (2024) [44] Egypt	9 studies of participants with varied CVD *N* = 365 in total	ISI, PSQI	Interventionist was not strictly reported		Significant improvements in ISI, and PSQI across the sample. No analysis conducted according to disease type. Recommend further CBT research.
Retrospective analysis: Conley et al. (2023) [27] USA	Participants of HeartSleep study	ISI			Most participants of CBT-I group had improving trajectories over 1 year. CBT-I did not cause significant results when adjusted for characteristics of symptom group- research needed for why.
Secondary analysis: Conley et al. 2022 [46]; USA	Participants of HeartSleep study	ISI, PSQI			No significant changes in self-care confidence between groups. Self-care improvement= PSQI score reduction. Recommend education+ psychology.
Literature review: Conley and Redeker 2016 [32]; USA	9 studies *N* = 407 Older adults with comorbid insomnia	Self-reported sleep quality			CBT-I improved morbidity, symptom burden, functional performance, and QoL. Studies with follow-up= result longevity. Recommend research into which approach is most effective.
Secondary analysis: Doering et al. (2016) [34] USA	*N* = 53 Cardiac surgery patients (CAB, valve replace)	PSQI	CBT vs. usual post-op care 8-week program Individual, 1 h sessions, face-to- face (F2F)	Sessions delivered in patient home	No differences in overall sleep quality between groups, pain improved. Recommend consideration of how CBT combat mental effect on sleep.
RCT: Gheiasi et al. (2024) [35] Iran	*N* = 90 Open heart surgery patients	PSQI	CBT vs. usual post-op care 10-week program, option for + 2 Individual, 1 h sessions, F2F	Location varied- occurred at agreed time and place by patient and interventionist	Significant improvements of PSQI in both groups but CBT groups higher. Educational, behavioural, and cognitive components improve sleep. Recommend CBT training.
RCT: Harris et al. (2018) [40] USA	*N* = 23 NYHA class I-III	ISI, PSQI	BBTI vs. sleep monitoring control group 4-week program designed by Buysse Individual, varying length, F2F and telephone calls bi-weekly	Community (location not reported)	BBTI causes significant improvements in sleep outcome measures + symptom reduction. Brief and individualised format accommodating. Recommend research into telephone-based interventions for maximal benefit.
Pilot RCT: Harris et al. (2019) [41] USA	*N* = 21 NYHA class I-III	ISI, PSQI, treatment fidelity checklist	BBTI vs. sleep monitoring control Intervention as above	Community (location not reported)	BBTI causes significant improvements in sleep outcome measures, low remission of insomnia symptoms, high treatment response. Protocol =treatment fidelity, educated professionals can deliver with min training. Recommend larger study.
Pre-post study: Harris et al. (2019) [41] USA	*N* = 47 Diagnosed with insomnia and CVD	ISI, self-reported sleep quality rating	CBT-I vs. Heart Healthy sleeping group (control) 6-week program Group, 90 min session, F2F	Community (location not reported)	ISI significantly improved, fewer anxiety, depression and insomnia symptoms. Recommend RCT with follow up to confirm findings of the study.
Pilot RCT: Javaheri et al. (2020) [38] USA	*N* = 34 CHD+ insomnia diagnosed (previous MI or revascularisation surgery)	ISI	Web-based CBT (Go! To sleep) vs. general sleep education 6-week program Individual, 6–7 topics, interactive online program		Significant, moderate reduction in ISI in intervention group. Physical health improved (not significant). Feasible, high adherence and completion rate but difficult recruitment. Recommend use of cardiologist to assist recruitment.
Systematic review: Kwekkeboom and Bratzke 2016 [47]; USA	13 articles, 9 studies *N* = 722 Adults with CHF and/or COPD	PSQI			7/9 reported beneficial effects of CBT strategies. Relaxation, meditation, and guided imagery or combo enhance improvements control. Results varied. Recommend research on duration, type, best way to improve relief in advanced symptoms and affecting variables.
Observational study: O’Connell et al. 2022 [45]	*N* = 112 from HeartSleep study	ISI, PSQI	Questionnaires and self-reported sleep outcome measures to gain data. 6-month follow-up data measured during pandemic.		Participants maintained/improved ISI, no significance between groups, both have protective effect. Sleep quality and duration not sustained. Recommend research of benefits of self-management.
RCT: O’Connell et al. (2023) [31] USA	*N* = 91 NYHA class I-II	Provider training, treatment delivery, receipt, enactment	NIH Behaviour Change Consortium (BBC) framework applied to HeartSleep study RCT		Suitable training, 93–95% of CBT protocol covered, group sessions highly attended and supported fidelity. Recommend studies in clinical environments to test implementation.
Pilot RCT: Redeker et al. 2015 [25]; USA	*N* = 48 NYHA class I-III	ISI, PSQI	CBT-I vs. attention control group 8-week program Group, 1 h session bi-week, F2F	Community (location not reported) Intervention- Clinical Psychologist	Moderate-large effect on CBT-I ISI CBT-I is feasible and effective. None deteriorated in CBT group. Research needed for sustained effects, survival rate.
Pilot RCT: Redeker et al. 2019 [28]; USA	*N* = 51 NYHA class II-IIII	ISI, PSQI	As above	As above	CBT improves dysfunctional thoughts causing improvements in ISI. Sustained, seen best at 6 month follow-up. Recommend CBT should include cognitive therapy.
Secondary analysis of RCT: Redeker et al. (2020) [48] USA	As above	ISI, PSQI	As above	As above	Evidence of relations between some biomarkers and sleep disturbance- research needed for CBT impact.
RCT: Redeker et al. (2022) [26] USA	*N* = 175 NYHA class II-III	ISI, PSQI	CBT- (Healthy Sleep) vs. HF self-management (Healthy Hearts) 8-week program Group, 1 h sessions bi-weekly, F2F	Community (location not reported) Intervention- PhD-prepared psychiatric nurse practitioner trained in CBT-I.	Significant reduction in ISI at 6/12 months. Improvements in HS group 2x as large, both groups had poor sleep at 12month. Recommend stepped care approach with web, app, tele-health.
Secondary analysis: Redeker et al. 2023 [29]; USA	Participants of HeartSleep study	ISI	As above	As above	Significant negative associations in insomnia severity on cognitive ability. Recommend identification of HF patients at risk of cognitive decline to do CBT.
RCT: Rybarczy k et al. (2011) [33] USA	*N* = 92 33 participants with CAD	PSQI	Book CBT-I vs. multimedia CBT-I Based on Morin protocol 8-week program Book= mailed material Multimedia= mailed material, 2 videotapes of CBT-I, audiotape.	Support from psychology graduate students	Multimedia CBT-I= larger but modest improvements in all sleep outcome measures (significant) over book. Both groups maintained over 1 year. Self-help CBT-I cost-effective. Recommend testing at primary care setting as optimal setting.
RCT and cross-sectional analysis: Schiele 2021 [42]; USA	*N* = 30 in study 1 NYHA class I-III *N* = 15 in study 2 HF but no insomnia	ISI, PSQI, BBTI treatment fidelity checklist	BBTI vs. sleep monitoring group 4-week program 2-week F2F, 2-week telephone. Buysse protocol	Community (location not reported) Advanced-level doctoral students	81% completed BBTI, format flexible, accommodating. Significant improvements in all sleep outcomes in BBTI group, low none-response rate. Higher health perception score=poorer sleep. Recommend testing of virtual form.
RCT: Siebmann s et al. (2021) [36] Sweden	*N* = 48 Verified CVD diagnosis and insomnia	ISI, adherence	Internet based CBT vs. self-study program 9-week program	Support from registered nurse trained in CBT-I support	Clinically significant (mod) effect of I-CBTI. Maintained at 6 month follow-up. Adherence= reduction in median ISI. Recommend consideration for nurse delivery, larger sample and CVD outcome.
Secondary analysis: Skúladótti r et al. (2024) [37] Sweden	*N* = 127 Symptomatic paroxysmal AF	ISI	Internet based CBT vs. education 10-week program	Therapist-led	Significant reduction and between group differences in ISI. Maintained at 3 month follow-up. Recommend exposure-based CBT to fill void between treatment and lifestyle change interventions.
RCT: Skúladótti r et al. (2024) [39] USA	N:130 Confirmed MI at least 2months ago	Sleep duration, efficiency and wakefulness to determine sleep quality	CBT vs. heart disease education 8-week intervention Group, 1 h teleconference sessions	Community (location not reported) Trained MBCT-Brief facilitator	Significant improvement in sleep duration in CBT, not control. Continuing mindfulness practice not associated in improvement at 6 month follow-up. Adherence better in control, high satisfaction. Recommend evidence for achieving result longevity.
Pre-test post-test: Stallman et al. (2023) [43] Australia	*N* = 72 In-patients with cardiac disorder (CHD, arrhythmia, HF, HTN, etc.).	Subjective sleep quality	Care Collaborate Connect (CCC) One session, one follow-up Individual, mean duration 37.5 min, F2F	In-patient hospital wards	Reliable improvements of sleep duration at 1 month follow-up. 43% found intervention effective, 21%,15% found not helpful.14% readmitted within 1 month, consistent with local rate of readmission. High acceptability, recommend efficacy studies for CCC.

### Outcome measures

Many of the results included key outcome measures related to insomnia severity and the patients’ perceptions regarding their sleep quality. The primary outcome measures used to inform this review were improvements in the Insomnia Severity Index (ISI) and self-reported sleep quality, often quantified by the Pittsburgh Sleep Quality Index (PSQI). Subjective measures such as the Dysfunctional Beliefs and Attitudes about Sleep (DBAS) and the Sleep Disturbances Questionnaire (SDQ) were also considered to provide insights into patient beliefs and attitudes regarding their sleep. Secondary outcome measures assessed in this report included treatment fidelity checklists to explore patient perceptions and acceptability of the intervention delivered. Fatigue, quality of life and mental health outcome measures were commonly studied as secondary outcome measures, but the results of these have not been analysed due to the purpose of this review.

### Included studies

Randomised controlled trials (RCTs) were the most common amongst the results (8/24; 33%), alongside secondary analysis of an RCT (6/24; 25%) and pilot RCTs (4/24; 16%). Two pre-test, post-test studies (2/24; 8%), a mixed-method study, an observational study, two systematic reviews and a literature review stood for the remaining results, providing a variety of quantitative and qualitative data. Almost all of the trials were conducted in America (13/15; 87%), with the remaining trials conducted in Sweden (2/15), Iran (1/15) and Australia (1/15). HF patients were the most prevalent comorbidity group, followed by coronary artery disease (CAD) and/or primary diagnosis of CVD, patients undergoing cardiac surgery, including revascularisation or valve replacements, MI and atrial fibrillation (AF). 14 trials tested independent interventions.

### Group cognitive behavioural therapy (CBT)

12 articles, consisting of trials and data analysis, studied the results produced when the CBT intervention incorporated group meetings. The mean programme duration was seven and a half weeks, with the mean intervention duration lasting 67.5 min. Two studies reported statistically significant (*p*< 0.05) reductions in ISI [25, 26], with Redeker et al. [26] finding improvements twice as large in the intervention group. In comparison, Conley et al. [27] found that participants with severe and non-improving symptoms received no statistically significant benefits from CBT compared to usual care. Alongside larger improvements in comparison to the control groups, Redeker [26] also found that effects were sustained at six months post-treatment, supported by further secondary analysis reports published by Redeker, which focused on sleep-cognitions [28] and cognitive functions [29]. One article did not conduct follow-up assessments [30], yet the author recognised the need to understand the sustained effects of group CBT. O’Connell et al. [31] reported that participants from the HeartSleep study maintained or improved post-treatment levels of insomnia severity during the pandemic, lasting up to four years, with follow-up appointments demonstrating improved longevity in results [32]. Group CBT also had the highest reported treatment fidelity, with strategies recommended to be used as guidelines to implement as a new intervention.

### Individualised CBT

Three studies tested individualised CBT interventions on patients through a variety of modality deliverance, including multimedia-based CBT [33]. The use of CBT delivered through paper or video material not only improved sleep measures, which were maintained post-treatment, but also enhanced self-care [33]. Doering et al. [34] reported no differences in sleep quality between the intervention (CBT) and control group, who received usual care. In comparison, Gheiasi et al. [35] found statistically significant improvements in PSQI scores between the control and intervention groups (*p*= 0.0001). Both samples received similar intervention in the form of in-person, one-hour sessions, with the only notable difference being a two-week discrepancy in intervention delivery. Therefore, it may be reasonable to assume that a ten-week programme can be used as guidance for minimum treatment duration [35], in comparison to the eight-week programme [34].

### Internet-based CBT

Four articles utilised internet-based CBT, all producing results in favour of its use. The mean programme duration was 8.6 weeks, all based on module or topic content. Despite slight variations in the content of the intervention, reductions in ISI were reported both post-treatment and at follow-ups. Siebmanns [36] reported that reductions in median ISI score from baseline to post-treatment in the experiment group were statistically significant (*p*= 0.002), but results did not remain significant at a 6-month follow-up. Skúladóttir et al. [37] found that improvements in ISI from baseline to the three-month follow-up were significant compared to the control group (*p* = 0.032), possibly demonstrating concerns regarding the sustainability of improvements past three months. Despite this, alongside clinically relevant improvements in ISI (*p*= 0.03), this method also boasted a high adherence and completion rate amongst older adults [38].

### Mindfulness-based CBT (MCBT)

One article explored the impact and adherence of a mindfulness-based CBT programme on women after suffering an MI [39]. Reductions in sleep duration were statistically significant (*p*= 0.038), but continuation of mindfulness practice was not associated with any changes in sleep quality or duration at the six-month follow-up [39]. Adherence was rated fair to good, but was better in the control group due to lower time commitment and individuality [39].

#### Brief behavioural treatment for insomnia (BBTI)

Three RCTs utilised a BBTI intervention compared to a sleep monitoring control group in patients with HF. Results produced by Harris [40] found statistically significant improvements in ISI (0.003) and PSQI scores (0.005), with the results replicated in the pilot RCT published in 2019 [41], whereas Schiele [42] was unable to conclude significant results due to a small sample size. Similar to results published when using CBT, participants experienced insomnia symptom remission as low as 6.3% of the sample size [40, 41]. Both Schiele [42] and Harris et al. [41] concluded BBTI to be a well-tolerated, flexible method of delivery when used in the Buysse format. The incorporation of telehealth and the need for minimal training by the professionals delivering the intervention enhanced the treatment fidelity by accommodating to the participants’ needs [41].

### Psychological interventions

One article employed the Care Collaborate Connect intervention to measure subjective sleep quantity and quality through questionnaires. Stallman et al. [43] found that 43% of the participants found the low-intensity psychological intervention effective, with only 21% of cardiac surgery patients finding it helpful. 8.3% of participants had positive, reliable improvements at the one-month follow-up, but 14% were readmitted to the hospital before the follow-up, consistent with the usual, local rate of readmission [43].

## Discussion

This scoping review aimed to collate and synthesise current published literature regarding the use of cognitive and behavioural sleep interventions. This review included 24 results, including 16 clinical studies, six secondary data analyses, and three evidence reviews. The studies within this review suggest that the use of behaviour change interventions is overall effective in reducing the severity of insomnia, improving sleep quality and changing dysfunctional thoughts about sleep [44]. Many authors endorsing the use of behaviour change interventions also considered treatment fidelity, underscoring the importance of evaluating the clinical efficacy of a BC intervention, but also the participant acceptability and engagement to enhance intervention delivery and stakeholder need alignment. To apply the findings to clinical practice, specific intervention modalities and formats must be considered and tested against large and robust populations.

Of the 24 results, 19 tested and analysed CBT, therefore determining this to be the most consistently researched intervention within this scoping review. Modules commonly covered within the CBT interventions included sleep hygiene education by encouraging consistent sleep schedules and bedtime routines, stimulus control and sleep restriction [13, 14]. Physical relaxation techniques are also taught, alongside the ability to recognise and prevent poor habit relapse (Stakeholders confirmed that sleep hygiene was ineffective as a standalone therapy). Alongside improvements in sleep outcome measures, CBT was also seen to enhance self-care and management through education and support to maximise independence [33, 45, 46]. The detailed but complex methodology encompassed within CBT enhances the interventions’ ability to produce similar improvements across patients with varying disease severity. The incorporation of relaxation, meditation and guided imagery components within CBT has been well endorsed, but the extent to which each component of CBT has the largest effect on symptom trajectory is yet to be explored [47]. In line with this, the populations represented across the evidence base span a wide clinical spectrum, ranging from stable HF populations (New York Heart Association [NYHA] class I) to highly symptomatic individuals (NYHA class IV–V). Adjustment for clinical and demographic heterogeneity in the evaluation of cognitive behavioural therapy for insomnia (CBT-I) outcomes was undertaken in only one study [27]. Consequently, the ability to generalise findings across studies is limited, particularly when extrapolating results from populations including cardiac surgery, CAD, and MI patients to the wider CR population, or when attempting to determine which intervention confers the greatest effect. In addition, the CR programme settings and exercise prescriptions varied substantially between studies, reflecting both the underlying cardiac condition and alignment with country-specific national guidelines. This heterogeneity highlights a key research gap regarding how variation in CR delivery models may influence the feasibility and implementation of sleep management interventions within routine CR services.

Group-based CBT is consistently supported as an effective mode of delivery, owing to its association with clinically meaningful and sustained improvements in insomnia outcomes, alongside high treatment fidelity. Group delivery uniquely facilitates peer support, shared learning, and normalisation of sleep difficulties; elements that align well with the established group-based structure of CR programmes. The use of scheduled sessions promotes behavioural regularity and adherence, while also offering efficiencies in service delivery by optimising limited healthcare resources and staffing capacity. However, clinicians should carefully consider patient suitability for group-based CBT, as treatment effects may be attenuated in individuals with severe or treatment-resistant insomnia [25]. Accordingly, structured screening procedures are essential, alongside appropriately trained interventionists to ensure effective facilitation and maintenance of group cohesion.

The effectiveness of CBT is not uniform across the patient groups, with some studies indicating a reduced benefit in participants with severe or treatment-resistant insomnia. Therefore, tailored intervention types, alongside patient screening and stratification, are essential considerations for rehabilitation. Web-based CBT is a particularly well-supported method in improving subjective sleep outcome measures, specifically the ISI [26]. Skúladóttir et al. [37] determined that flexible methods of delivery, such as online programmes, allowed CBT to fill the void between medical treatment and lifestyle interventions by utilising exposure-based therapy to address psychological, behavioural and social influences of symptoms. High completion rates suggest acceptability, a key consideration when clinicians attempt to integrate a new treatment modality.

Interactive web-based programmes also boasted high adherence [34, 38], but there are indications of concerns regarding the involvement of technology, such as poor computer skills or difficulty adapting to an online programme [34]. When combined, Spruill et al. [39] discovered that group sessions delivered online resulted in significant improvements in sleep duration and efficacy but had lower treatment adherence than the control group due to the increased time commitment. Considering this, it has been widely recommended by authors across the evidence base for stakeholders to consider the promising results of CBT benefits, but more research needs to be done into the use of telehealth regarding the flexibility and accommodation for patients, particularly those who are highly burdened (41; 48).

Currently, exploration into the effect of BBTI in CVD patients is underdeveloped, as evidenced by the low representation within this review. Harris et al. [40] reported that BBTI is an accommodating, well-tolerated format of behaviour change, which produces significant improvements in outcome measures and reductions in symptom burden of HF patients. The use of an established protocol and incorporation of physical and telehealth delivery ensured adaptability for dependent participants [40], evident in high completion rates and low no-treatment-response rates [42]. To further endorse the use of BBTI, Harris et al. [40] concluded that this intervention can be delivered by clinicians with minimal training, conflicting with Gheiasi’s [33] recommendation for clinicians to receive specialised training to deliver CBT. Future studies may consider testing the Buysee protocol on larger samples [41] to determine the potential scope of impact of BBTI. Furthermore, recommendations have been made for research to determine how telehealth or virtual forms of BBTI may be of benefit to CVD patients [40, 41].

Within this review, heterogeneity in intervention protocols introduces uncertainty regarding the optimal standardised approach to implementation. While all delivery formats demonstrated clinically meaningful improvements, this variability underscores the need for a more tailored approach to screening, stratification, and treatment, whereby patients’ symptoms are addressed according to individual clinical need. Core components of behaviour change interventions, as outlined previously, should therefore be prioritised to ensure that delivery formats can be effectively embedded within existing CR structures. As demonstrated in Table 1, behaviour change interventions are currently delivered predominantly by trained nurses, psychologists, and therapists; however, the development of standardised protocols and clearly defined referral pathways presents opportunities to broaden the workforce through targeted training of additional health professionals. Potential barriers to implementation, including time constraints, patient engagement, and digital literacy, must be acknowledged and proactively addressed. To achieve sustained benefit, stakeholders should advocate for the long-term integration of structured behaviour change interventions within CR services, to enhance both service efficiency and patient outcomes.

### Previous research

The findings of this review support the findings of Hertenstein et al. [49], who determined that, to a large extent, CBT is favoured as the treatment for insomnia severity in patients with mental disorders and comorbid insomnia. In line with this, a reduction in insomnia directly after treatment and at six-month follow-ups following CBT is explained as a commonly reported effect, most likely due to the behaviour change strategies implemented [49]. Furthermore, this systematic review also determined large heterogeneity in research regarding insomnia treatment, in this case, a dominant depression-focused population [49]. Kaar et al. [50] also explored the use of behaviour change interventions on sleep, alongside psychosocial symptoms and physical health behaviours. Through conclusions made by analysing existing literature, Kaar et al. [50] demonstrates how behaviour change interventions, including CBT, mindfulness-based interventions and motivational interviewing, can be used to improve sleep, quality of life and physical activity. Kaar et al.’s [50] recommendations of blended collaborative care for patients at risk of or living with CVD can be used in conjunction with this review to place an emphasis on the need for health behaviour change interventions to be implemented into cardiac care to enhance outcomes.

Although there is an overwhelming demand for an increase in homogeneity and standardisation between sleep treatment research, Winter et al. [51], within their narrative review, hypothesised that research is unable to comprehend a modern and ever-evolving treatment base. This has been further emphasised by the advancements made by technology and, therefore, the need for traditional behaviour change models and interventions, such as CBT, to be re-conceptualised to accommodate the advancements [51]. Amirova et al. [52]performed a systematic review and meta-analysis with the intention of collating the barriers and enablers to HF patients, informing effective behaviour change interventions. Understanding the contextual barriers, such as symptom burden, and encompassing modifiable enablers, such as a patient’s knowledge and belief in consequences, is essential to inform the use of behaviour change interventions [52]. To accommodate this, more research needs to be published to address the barriers to implementation of behaviour change into routine care and how technology may be used to enhance the enablers.

### Gaps in the literature

There remains a notable paucity of literature examining behaviour change interventions delivered to participants actively enrolled in, and routinely attending, cardiac rehabilitation (CR) programmes. Although several included studies recruited participants with cardiac conditions commonly eligible for, or referred to, CR services, only one study [43] explicitly involved patients engaged in inpatient CR, and none examined how CR participation may have influenced intervention delivery or outcomes. This represents a clear evidence gap regarding the implementation of sleep and behaviour change interventions within real-world CR environments, despite strong conceptual and guideline alignment with CR models of care.

Notwithstanding this gap, the intervention components and delivery principles described across the included studies broadly reflect existing CR guidelines, suggesting that integration within CR settings is potentially feasible. Supporting this translational potential, recent field-level calls to action [53, 54] have advocated for the systematic inclusion of sleep assessment and management within rehabilitation programmes, including CR, to enhance programme effectiveness and patient-centred care. Furthermore, literature suggests that exercise interventions alone in CR may be insufficient to address sleep disturbances within CR services and may even exert adverse effects if prescribed without adequate consideration of the contribution of sleep to overall cardiovascular burden [55, 56], emphasising the need for individualised sleep management pathways like the CBT approaches. Therefore, dedicated studies involving CR-enrolled populations are urgently required to evaluate acceptability, engagement, and effectiveness of the sleep management pathways within routine CR service delivery.

Also, when considering study populations, patients with HF were evidently the most represented group. However, there was notable heterogeneity in the dataset regarding the disease classification and symptom burden. This review cannot definitively conclude that evidence regarding behaviour change for CVD patients is concentrated towards HF patients, but it may highlight the need for further research into optimal format depending on morbidity and symptom profiles. Questions may be raised regarding the interdisciplinary integration of behaviour change interventions, as there is a clear gap in the role of the multidisciplinary team in delivering or monitoring the interventions. The multifactorial nature of behaviour changes places a demand for the expertise of a team of varied health professionals, but there is no evidence of allied health professionals’ involvement with the interventions.

Many published trials reporting on the use of CBT and BBTI are conducted in America, presenting challenges when attempting to generalise results to worldwide healthcare systems. The heterogeneity of the study intervention designs presents a challenge for evidence synthesis and limits result comparability between the studies. While results of clinical trials are conclusive, previous systematic and literature reviews within this review have been unable to make clear recommendations regarding the most clinically and cost-effective intervention protocol. This demonstrates the clear need for standardisation in protocol and follow-up timeframes with stratified outcomes to first personalise the treatment approach according to patient characteristics and secondly to improve the ease of integrating behaviour change interventions into routine cardiac care. A recommendation can be made for future research to also further consider the cost effectiveness of and engagement of participants in a BC intervention, as conclusions cannot be made from the analysis within this review.

### Strengths and limitations

This scoping review can be used in conjunction with existing literature regarding the use of different BC techniques for patients specifically with CVD. This review highlights the potential benefits and feasibility of utilising BC interventions for patients living with CVD, with the intention of inspiring further consideration into how they may be implemented into an established care programme, such as CR. In terms of strengths, this review utilised a methodology guided by frameworks and established clear inclusion and exclusion criteria to inform result selection. Utilising multiple medical databases provided access to a high number of articles, which is beneficial when attempting to meet the aim of collating valuable and insightful data. Yet, due to the complexity of this topic and independent researcher screening the results, it is reasonable to assume some relevant articles may have been missed. The selection of databases may have also contributed to the relatively small sample size for a scoping review, first due to the limited dataset available within PsycINFO and CINAHL+. Furthermore, conducting a search across the Cochrane library produced a large amount of unpublished clinical trials, resulting in 32 articles and the results of those that may have been relevant being omitted from this scoping review.

As a scoping review, quality appraisal assessment was not conducted. Although including extensive heterogeneous data has provided opportunity for a comprehensive understanding of the topic, the lack of opportunity for generalisabilty cannot be disregarded. If repeated, future authors may consider the appropriate statistical analysis of the data collected to quantify the heterogeneity to produce increased robust conclusions. Furthermore, recommendations taken from evidence which have not been critical appraised should always be considered with caution and be combined with results interepreted from uniform, standardised trial.

## Conclusions

This scoping review provides a synthesis of the results found and accessible relating to the use of behaviour change interventions for patients living with CVD who also experience insomnia and poor sleep quality. It can be concluded that, from this review, flexible treatment modalities such as web-based CBT and BBTI may be the most suitable intervention to be incorporated for CVD patients with potential feasibility of implementation across the CR programmes, due to the improvements in sleep outcome measures and treatment fidelity. Considering this, key gaps in evidence including lack of homogenous data and comparison between standardised intervention length and duration and session content needs to be considered. Rigorous study design utilising large and diverse samples, including participants currently undergoing a CR programme, will inform the clinical efficacy of a BC intervention and how to address current barriers to implementation.

## Supplementary Information

Below is the link to the electronic supplementary material.

Supplementary Material 1 (DOCX 19.2 KB)

## Data Availability

My manuscript has no associated data.
