# Supplementary material for: Could behaviour change interventions be incorporated into cardiac rehabilitation programmes for insomnia and poor sleep quality management? A scoping review
Source: Sleep Breath. 2026 May 23;30(3):169. doi: 10.1007/s11325-026-03707-x (PMC13198456; doi:10.1007/s11325-026-03707-x)
Supplement: Supplementary file 1 — Supplementary Material 1 (DOCX 19.2 KB) [file 11325_2026_3707_MOESM1_ESM.docx]

Appendix A: Scoping Review Search Strings and Terms

Medline

|  | Population | Intervention | Outcome |
| --- | --- | --- | --- |
| Subject Heading | “*cardiovascular disease”*  *“cvd”*  *“heart or cardiac or coronary heart disease”* | *“behaviour change models”,*  *“behaviour change theory”*,  *“behaviour change wheel”*,  *“brief behavioural therapy for insomnia”*  *“cognitive behavioral therapy”*  *“cbt”*  *“cognitive behavioural therapy”* | *“sleep quality or quality of sleep”*,  *“sleep improvement”*  *“sleep quality”*,  *“sleep quantitity”*,  *“sleep duration”*  *“hours of sleep”*  *“sleep quantity”*  *“total sleep time*  *“sleep amount”* |
| MeSH Heading | “Cardiovascular Diseases” | “Transtheoretical Model” |  |

Date range initial search 01/01/2010- 24/03/2025

Date range second search 24/03/2025- 29/03/2026

Limiters- published in English, Human Participants, Peer Reviewed Publications

((cardiovascular disease or cvd or heart or cardiac or coronary heart disease) OR ((MH "Cardiovascular Diseases"))) AND ((behaviour change models) OR (behaviour change theory) OR (behaviour change wheel) OR (brief behavioral therapy for insomnia) OR (cognitive behavioral therapy or cbt or cognitive behavioural therapy) OR ((MH "Transtheoretical Model") OR "behaviour change models")) AND ((sleep quality or quality of sleep) OR (sleep improvement and sleep quality) OR (sleep quantity) OR (sleep duration or hours of sleep or sleep quantity or total sleep time or sleep amount))

APA PscyInfo

|  | Population | Intervention | Outcome |
| --- | --- | --- | --- |
| Subject Heading | “*cardiovascular disease”*  *“cvd”*  *“heart or cardiac or coronary heart disease”* | *“behaviour change models”,*  *“behaviour change theory”*,  *“behaviour change wheel”*,  *“brief behavioural therapy for insomnia”*  *“cognitive behavioral therapy”*  *“cbt”*  *“cognitive behavioural therapy”* | *“sleep quality or quality of sleep”*,  *“sleep improvement”*  *“sleep quality”*,  *“sleep quantitity”*,  *“sleep duration”*  *“hours of sleep”*  *“sleep quantity”*  *“total sleep time*  *“sleep amount”* |
| MeSH Heading | “cardiovascular diseases” | “Transtheroetical model” |  |

Date range initial search 01/01/2010- 24/03/2025

Date range second search 24/03/2025- 29/03/2026

Limiters- published in English, Human Participants, Peer Reviewed Publications

((cardiovascular disease or cvd or heart or cardiac or coronary heart disease) OR ((MH "Cardiovascular Diseases"))) AND ((behaviour change models) OR (behaviour change theory) OR (behaviour change wheel) OR (brief behavioral therapy for insomnia) OR (cognitive behavioral therapy or cbt or cognitive behavioural therapy) OR ((MH "Transtheoretical Model") OR "behaviour change models")) AND ((sleep quality or quality of sleep) OR (sleep improvement and sleep quality) OR (sleep quantity) OR (sleep duration or hours of sleep or sleep quantity or total sleep time or sleep amount))

Cinhal+

|  | Population | Intervention | Outcome |
| --- | --- | --- | --- |
| Subject Heading | “*cardiovascular disease”*  *“cvd”*  *“heart or cardiac or coronary heart disease”* | *“behaviour change models”,*  *“behaviour change theory”*,  *“behaviour change wheel”*,  *“brief behavioural therapy for insomnia”*  *“cognitive behavioral therapy”*  *“cbt”*  *“cognitive behavioural therapy”* | *“sleep quality or quality of sleep”*,  *“sleep improvement and sleep quality”*,  *“sleep quantitity”*,  *“sleep duration or hours of sleep or sleep quantity or total sleep time or sleep amount”* |
| CINAHL Heading | “Cardiovascular Diseases” | “Transtheoretical Model” |  |

Date range initial search 01/01/2010- 24/03/2025

Date range second search 24/03/2025- 29/03/2026

Limiters- published in English, Human Participants, Peer Reviewed Publications

((cardiovascular disease or cvd or heart or cardiac or coronary heart disease) OR ((MH "Cardiovascular Diseases"))) AND ((behaviour change models) OR (behaviour change theory) OR (behaviour change wheel) OR (brief behavioral therapy for insomnia) OR (cognitive behavioral therapy or cbt or cognitive behavioural therapy) OR ((MH "Transtheoretical Model") OR "behaviour change models")) AND ((sleep quality or quality of sleep) OR (sleep improvement and sleep quality) OR (sleep quantity) OR (sleep duration or hours of sleep or sleep quantity or total sleep time or sleep amount))

Cochrane Library

|  | Population | Intervention | Outcome |
| --- | --- | --- | --- |
| Subject Heading | “*cardiovascular disease”*  *“cvd”*  *“heart or cardiac or coronary heart disease”* | *“behaviour change models”,*  *“behaviour change theory”*,  *“behaviour change wheel”*,  *“brief behavioural therapy for insomnia”*  *“cognitive behavioral therapy”*  *“cbt”*  *“cognitive behavioural therapy”* | *“sleep quality or quality of sleep”*,  *“sleep improvement and sleep quality”*,  *“sleep quantitity”*,  *“sleep duration or hours of sleep or sleep quantity or total sleep time or sleep amount”* |
| MeSH Heading | “cardiovascular diseases” |  |  |

Date range initial search 01/01/2010- 24/03/2025

Date range second search 24/03/2025- 29/03/2026

Limiters- published in English, Human Participants, Peer Reviewed Publications

((cardiovascular disease or cvd or heart or cardiac or coronary heart disease) OR ((MH "Cardiovascular Diseases"))) AND ((behaviour change models) OR (behaviour change theory) OR (behaviour change wheel) OR (brief behavioral therapy for insomnia) OR (cognitive behavioral therapy or cbt or cognitive behavioural therapy) OR ((MH "Transtheoretical Model") OR "behaviour change models")) AND ((sleep quality or quality of sleep) OR (sleep improvement and sleep quality) OR (sleep quantity) OR (sleep duration or hours of sleep or sleep quantity or total sleep time or sleep amount))
